# Ultra-Thin Wrinkled Carbon Sheet as an Anode Material of High-Power-Density Potassium-Ion Batteries

**DOI:** 10.3390/molecules27092973

**Published:** 2022-05-06

**Authors:** Boshi Cheng, Xing Li, Linhai Pan, Hongqiang Xu, Haojie Duan, Qian Wu, Bo Yin, Haiyong He

**Affiliations:** 1 School of Materials Science and Chemical Engineering, Ningbo University, Ningbo 315211, China; chengboshi@nimte.ac.cn; 2 Ningbo Institute of Materials Technology and Engineering, Chinese Academy of Sciences, Ningbo 315201, China; panlinhai@nimte.ac.cn (L.P.); xuhongqiang@nimte.ac.cn (H.X.); duanhaojie@nimte.ac.cn (H.D.); wuqian20@nimte.ac.cn (Q.W.); hehaiyong@nimte.ac.cn (H.H.)

**Keywords:** potassium-ion batteries, anode, two-dimensional, carbon sheet

## Abstract

Although K^+^ is readily inserted into graphite, the volume expansion of graphite of up to 60% upon the formation of KC_8_, together with its slow diffusion kinetics, prevent graphite from being used as an anode for potassium-ion batteries (PIBs). Soft carbon with low crystallinity and an incompact carbon structure can overcome these shortcomings of graphite. Here, ultra-thin two-dimensional (2D) wrinkled soft carbon sheets (USCs) are demonstrated to have high specific capacity, excellent rate capability, and outstanding reversibility. The wrinkles themselves prevent the dense stacking of micron-sized sheets and provide sufficient space to accommodate the volume change of USCs during the insertion/extraction of K^+^. The ultra-thin property reduces strain during the formation of K-C compounds, and further maintains structural stability. The wrinkles and heteroatoms also introduce abundant edge defects that can provide more active sites and shorten the K^+^ migration distance, improving reaction kinetics. The optimized USC_20−1_ electrode exhibits a reversible capacity of 151 mAh g^−1^ even at 6400 mA g^−1^, and excellent cyclic stability up to 2500 cycles at 1000 mA g^−1^. Such comprehensive electrochemical performance will accelerate the adoption of PIBs in electrical energy applications.

## 1. Introduction

After research and development in recent years, potassium-ion batteries (PIBs) are considered to be a promising new energy storage system that can replace lithium-ion batteries (LIBs) in a number of application scenarios [1,2]. Carbon-based anode materials, which are rich in raw materials, have excellent conductivity, and are environmentally friendly, [3,4,5] have been successfully commercialized for LIB anodes, and also show great application prospects in the field of PIBs [6,7,8]. Ju et al. adopted P and O co-doped graphene as a PIB anode material, and delivered a specific capacity of 165 mAh g^−1^ at 2000 mA g^−1^ [9]. However, in practical applications, the heteroatom doping strategy may slash the initial coulombic efficiency (ICE) and increase the voltage hysteresis of the electrode material [10,11]. It is well known that hard carbon has poor conductivity [12,13], as well as high discharge potential and low energy density when serving as an anode for PIBs [13]. In regard to the application requirements of PIBs, soft carbon is a better choice.

Soft carbon attracts much attention because of its low charge–discharge voltage and high specific capacity [6,14]. However, soft carbons are subject to huge expansion stress during the insertion of K^+^, which can lead to the collapse of the electrode [12,14,15]. Thus, designing structures with high stress tolerance is important for developing new electrode materials. The power density of soft carbon as an anode for PIBs also needs to be improved because of the slow diffusion kinetics of K^+^ in soft carbon.

To accommodate expansion stress, elastic carbon aerogel is expected to be an outstanding candidate for improving the structural stability of PIB anodes [12,13]. Up to now, various elastic carbon aerogels have been built by nano-carbons, such as carbon nanotubes, graphene, graphene oxide, and biomass-derived carbon [5,13,16]. Carbon aerogel materials show potential in the field of potassium storage because of their high surface area, marvelous mechanical strength, and high conductivity [5,12,16]. On the other hand, reducing the number of stacked layers in the c-axis direction of soft carbon, i.e., preparing materials with thin sheet-like structures, likewise reduces the strain of K^+^ insertion/extraction. In addition, an ultra-thin structure facilitates the insertion/extraction of K^+^ and maintains structural integrity more easily. Therefore, an ultra-thin skeletal structure can achieve an excellent rate of performance and offer a long service life when serving as an anode for PIBs.

Therefore, the template effect of melamine (MA) in the carbonization process was used to prepare ultra-thin two-dimensional (2D) wrinkled soft carbon sheets (USCs). The wrinkled morphology is beneficial to absorb expansion stress and shorten K^+^ migration distance during the electrochemical process, boosting the rate capability. Even at 6400 mA g^−1^, the reversible specific capacity of USC_20−1_ still exceeds 151 mAh g^−1^. USC_20−1_ also owns an ultra-long cycling life span, with a specific capacity of 137 mAh g^−1^ after 2500 cycles at 1000 mA g^−1^. Meanwhile, USC_10−1_ exhibits a high reversible capacity of up to 444 mAh g^−1^ at 25 mA g^−1^.

## 2. Results and Discussion

**Figure 1:**
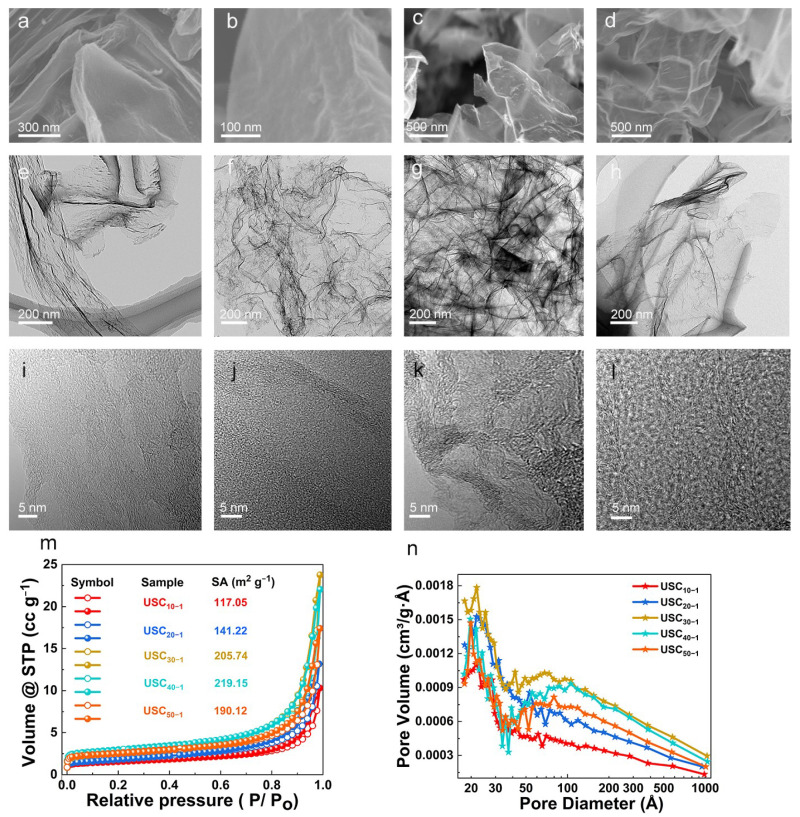
SEM and TEM images of (**a**,**e**,**i**) USC_20−1_, (**b**,**f**,**j**) USC_30−1_, (**c**,**g**,**k**) USC_40−1_ and (**d**,**h**,**l**) USC_50−1_. (**m**) N_2_ adsorption and desorption curves, and a (**n**) pore volume distribution diagram.

As shown in Figure 1a–d and Figure S1, USCs are made of wrinkled sheets of microscale diameter. Careful observation has revealed that the higher the proportion of MA and NH_4_Cl in the precursors, the thinner the resulting microscale sheets. The hemi-transparency property of the TEM and the enlarged SEM images suggest the ultra-thin nature of the sheets (Figure 1a–h). The wrinkled sheet structure not only prevents the dense stacking of electrode materials, but also alleviates the volume effect during charge and discharge. Most importantly, the ultra-thin property can reduce the strain during the insertion/extraction of K^+^, while the wrinkled property shortens the migration distance of K^+^ in the K-C compounds by offering abundant edge defects [13,15].

The HRTEM images (Figure 1i–l) clearly show the lattice fringes of carbon. The short-range ordered and long-range disordered structure of USCs not only accelerate the diffusion of alkali metal ions between the carbon interlayers, but also enhance the electron conductivity [15,17]. Therefore, USCs are an ideal choice for a PIB anode material. The specific surface area (SA) and pore structure of USCs were analyzed in detail by N_2_ adsorption and desorption tests (Figure 1m,n). Type IV isotherms with an H3 hysteresis loop demonstrate that all USCs have a mesoporous/macroporous structure [18,19]. Based on the Brunauer–Emmett–Teller (BET) method, the SAs of all the USCs were calculated and found to obey the following relationship: USC_10−1_ (117 m^2^ g^−1^) < USC_20−1_ (141 m^2^ g^−1^) < USC_30−1_ (205 m^2^ g^−1^) < USC_40−1_ (219 m^2^ g^−1^). Interestingly, the SA of USC_50−1_ decreased to 190 m^2^ g^−1^. This indicates that the ratio of MA in a reaction system can well regulate the SA of USCs, and the appropriate SA is conducive to increase the contact area between the electrode material and the electrolyte, as well as decrease the amount of generated solid electrolyte interphase (SEI) [9]. The pores around 20 nm can fully adsorb electrolyte due to capillary force, and serve as a K^+^ reservoir. This effectively shortens the ion migration distance and thus improves the rate performance of the electrode material.

**Figure 2:**
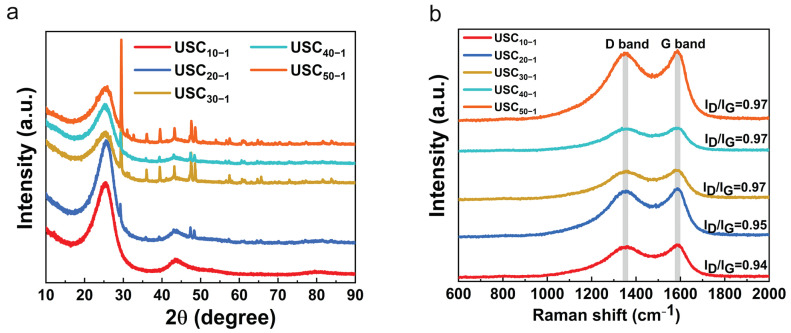
(**a**)XRD patterns and (**b**) Raman spectra of all samples.

As shown in Figure 2, XRD and Raman were used to analyze the structure of carbon. The XRD patterns of all samples in Figure 2a showed a slight shift and broadening of the (002) diffraction peak with increasing MA and NH_4_Cl content in the precursor, which means that the interlayer distance was enlarged and the stacked layers along the c-axis were reduced [20,21]. This is mainly caused by the following two reasons. First, MA and NH_4_Cl in the precursor act as a N source, and the introduction of heteroatoms promotes the expansion of interlayer spacing. Secondly, the template effect of MA is helpful to reduce the thickness of the prepared material. The enlarged interlayer spacing allows K^+^ to shuttle more easily within the material [22,23]. To summarize, the content of MA and NH_4_Cl in the precursor not only modulate the thickness of carbon sheet, which has also been confirmed by SEM and TEM, but also promote the expansion of interlayer spacing to some extent. There are two main peaks, namely the D band (1354.58 cm^−1^) and the G band (1587.6 cm^−1^) in the Raman spectra of the USCs in Figure 2b [17,24]. The G peak can be attributed to the vibrational peak of sp^2^ hybrid carbon, which reflects the crystallinity of the carbon material. The D peak (sp^3^ graphite configuration) reflects the disorder of the carbon material, mainly due to the introduction of heteroatoms and defects [25,26]. With the increase in MA and NH_4_Cl content in the precursor, the I_D_/I_G_ value of the carbon sheet gradually increased and finally reached a constant value (USC_10−1_ (0.94) < USC_20−1_ (0.95) < USC_30−1_ (0.97) = USC_40−1_ (0.97) = USC_50−1_ (0.97)).

**Figure 3:**
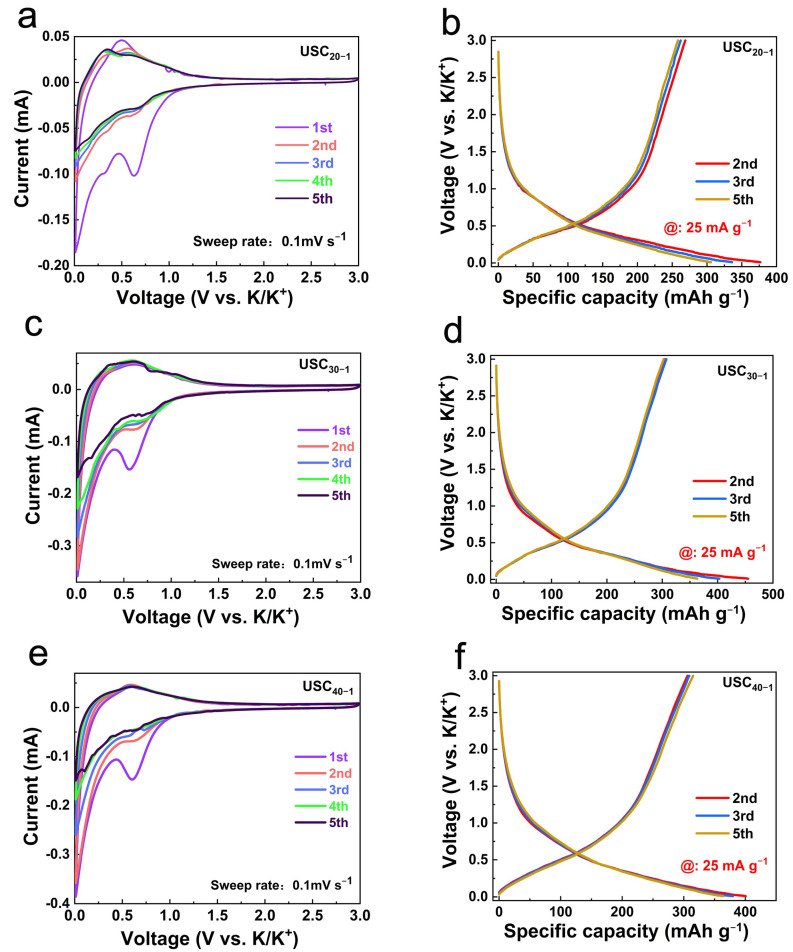
The CV curves at 0.1 mV s^−1^ scanning speed and charge–discharge curves at 25 mA g^−1^: (**a**,**b**) USC_20−1_, (**c**,**d**) USC_30−1_, (**e**,**f**) USC_40−1_.

As shown in Figure 3a,c,e and Figure S2a,c, a cyclic voltammetry (CV) test was used to study the electrochemical properties of all materials in the voltage range of 0.01 to 3.0 V at a scanning rate of 0.1 mV s^−1^. A strong irreversible reduction peak appeared near 0.6 V in the first cycle, which was related to the generation of solid electrolyte interphase (SEI) during the first discharge process [10,20]. The formation of SEI affects the initial coulombic efficiency (ICE) of the material, which is directly related to the SA of the carbon sheet. It is believed that the reduction peak of the USCs at ~0.65 V corresponded to K^+^ adsorption on the carbon sheet surface, while the reduction peak at ~0.16 V corresponded to K^+^ intercalation into the carbon sheet, and the oxidation peak at ~0.35 V was related to K^+^ de-intercalation from the carbon sheet [14,27,28,29]. The electrochemical process was also confirmed by the charge–discharge curves at the current density of 25 mA g^−1^ (Figure 3b,d,f and Figure S2b,d). With careful observation of the charge–discharge curves of the USCs, it can be seen that USC_40−1_ sample had the highest discharge specific capacity and a perfect coincidence of curves, which were inseparable from the material structure. USC_40−1_ had the largest SA, which exposed more active sites and increased the adsorption capacity in the high voltage region.

**Figure 4:**
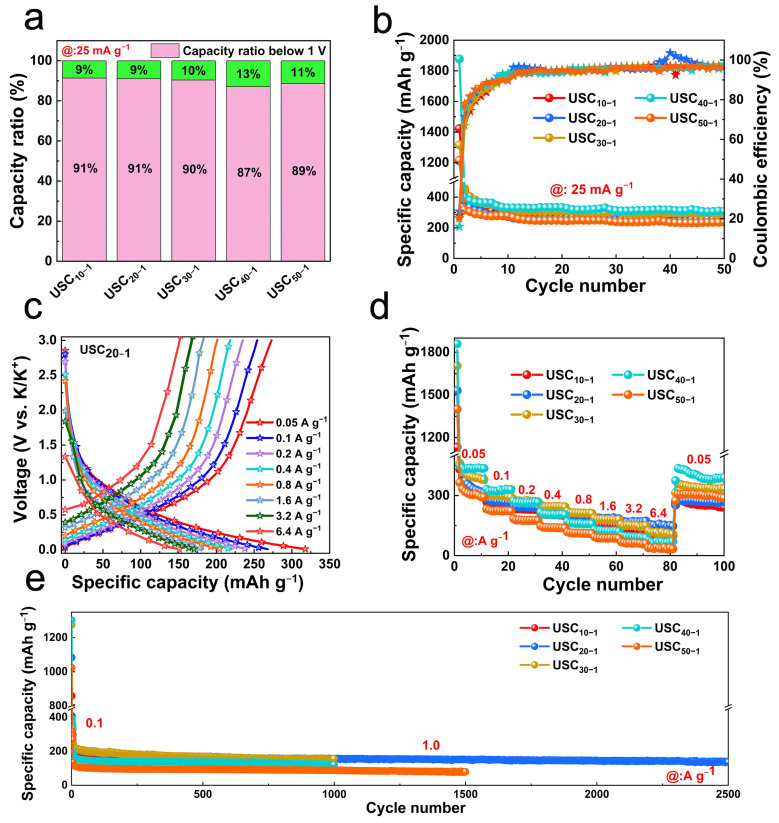
Electrochemical test. (**a**) Comparison of the capacities above and below 1 V at 25 mA g^−1^ of USCs; (**b**) cycle stability at 25 mA g^−1^; (**c**) charge and discharge curves of USC_20−1_ at different current densities; (**d**) rate performance; (**e**) cycle stability at 1.0 A g^−1^.

The potassium storage performance of the material in the voltage window of 0.01–3.0 V was tested by constant current charge and discharge. As shown in Figure 4a, the USC_40−1_ electrode displayed the highest specific capacity above 1 V, which can be attributed to the abundant heteroatom doping and the largest SA of USC_40−1_. Moreover, the specific capacity of USC_40−1_ remained at 308.7 mAh g^−1^ after 50 cycles at 25 mA g^−1^, superior to the other samples (Figure 4b). The charge and discharge curves of USC_20−1_ at different current densities are shown in Figure 4c. The reversible specific capacity was maintained at 151 mAh g^−1^, even at a high current density of 6.4 A g^−1^. The rate performances in Figure 4d show that USC_20−1_ had the highest discharge specific capacity at high current densities (>1.6 A g^−1^), while USC_40-1_ stood out at 0.05, 0.1 and 0.2 A g^−1^, and USC_30−1_ performed best at 0.4 and 0.8 A g^−1^. This means that this strategy can modulate the thickness of the electrode material for optimal performance depending on the application scenario. Such amazing rate performance is inseparable from the carbon structure. The enlarged interlayer spacing facilitates the transport of K^+^ and, at the same time, the wrinkled sheet structure prevents the dense stacking of 2D sheets and provides a buffer space for volume changes.

It is well known that service life is another important technical parameter for evaluating the prospects of electrode materials. No significant capacity degradation was observed for USCs after 1000 cycles at 1000 mA g^−1^. Furthermore, USC_20−1_ could still deliver a discharge specific capacity of 136.7 mA g^−1^ after 2500 cycles with an average decay rate of 0.017%. It is believed that USCs, as advanced anode materials for PIBs, will have broad application prospects.

**Figure 5:**
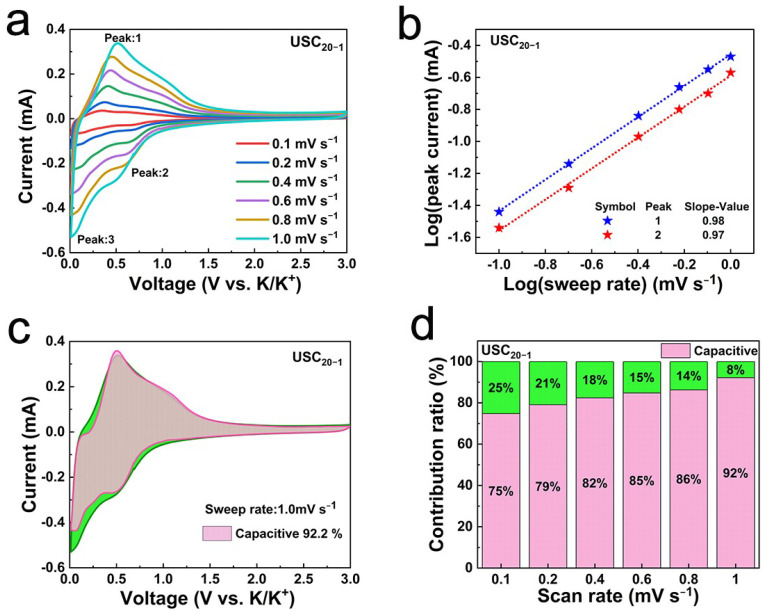
(**a**) CV curves at different scan rates; (**b**) linear fitting relationship between log i and log v at different redox peaks; (**c**) CV curves of electrode capacitance contributions at a scan rate of 1.0 mV s^−1^; (**d**) contribution ratio of pseudocapacitive response at different scan rates.

To further clarify the potassium storage kinetics of USCs, CV curves were recorded for all materials over the scan rate range of 0.1 to 1.0 mV s^−1^ in a voltage window of 0.01 to 3.0 V (Figure 5a and Figure S3a,e,i,m). With the stepwise improvement of scan rates, the response currents of the redox peaks of the USC_20−1_ electrode material increased rapidly, but the peak positions were only slightly shifted, indicating the fast redox reactions of USC_20−1_. According to the literature, there is a following relationship between peak current (i) and scanning rate (ν):$\;i={a*\nu }^{b}$, where a and b are constants [30,31]. When the b value is close to 0.5 or 1, the electrochemical behavior is dominated by diffusion or surface pseudocapacitance reactions, respectively [32,33]. As shown in Figure 5b and Figure S3, all the b values of USCs were close to 1.0, indicating that the potassium storage processes of the prepared electrode materials were controlled by capacitance behavior. The large SA of the wrinkled carbon sheets and the introduction of N doping sites were conducive to the adsorption of K^+^. The pseudo-capacitance contribution ratio can be calculated according to the following empirical formula:$i=k_{1}*v+k_{2}*v^{0.5}$ [34,35]. Where $k_{1}$ and $k_{2}$ are constants. As shown in Figure 5c and Figure S3, the fitted response current contributed by the capacitance process is represented by the pink area, and the total measured current at the scanning rate of 1.0 mV s^−1^ is represented by the green area. In general, the capacitance contribution ratios of all USCs electrodes increased with the improvements in scanning speed (Figure 5d and Figure S3d,h,l,p). However, the capacitance contribution ratios of USC_20−1_ at various scan rates were always higher than those of the other materials, which is in line with the best rate performance of USC_20−1_ at high current densities (>1.6 A g^−1^).

## 3. Experimental Section

Synthesis of USCs: First, certain amounts of MA and NH_4_Cl were weighed at a mass ratio of 4:1, and mixed thoroughly in an agate mortar (about 1 h). Then, different dosages (10 g, 20 g, 30 g, 40 g and 50 g) of the above prepared mixture were uniformly dispersed in a certain amount of N, N-dimethylformamide (DMF) solution by means of ultrasonication and stirring. Next, 1 g of pitch was added to the above-mentioned mixed solution and stirred at least for 6 h to fully dissolve the pitch. Subsequently, the solvent was completely volatilized by heating and then vacuum drying. The dried product was put into a tube furnace filled with argon for annealing after fully grinding. The annealing procedure was as follows: the sample was first heated to 200 °C at a heating rate of 1 °C min^−1^ and maintained for 2 h to soften the asphalt. Then, it was heated to 400 °C at 2 °C min^−1^ (kept for 2 h), and further heated to 600 °C at 4 °C min^−1^ (kept for 2 h). The final step was to heat the sample to 1100 °C at 5 °C min^−1^ (kept for 3 h). After natural cooling to room temperature, the final products were obtained and named as USC_10__−__1_, USC_20__−__1_, USC_30__−__1_, USC_40−1_ and USC_50−1_ according to the usage of the mixture of MA and NH_4_Cl.

Structural characterization: S4800 cold field emission scanning electron microscopy (SEM) and transmission electron microscopy (TEM Tecnai F20) were used to collect the microscopic morphology characteristics of the samples. A D8 ADVANCE DAVINCI X-ray powder diffractometer was used to collect the X-ray diffraction (XRD) spectra of the samples. A Confocal Raman Reflectance Microscope (Ram Enishaw Invia REFLEX) was used to accurately analyze the crystallinity and defects of the sample. The adsorption data of the multipoint Brunauer–Emmett–Teller (BET) method was used to calculate the specific surface area and pore size of the sample.

Preparation of anode electrode: At first, the active materials, sodium carboxymethyl cellulose (CMC-Na) and Super-P were mixed in a mass ratio of 8:1:1, and then stirred in deionized water to form a uniform viscous slurry. Subsequently, the paste was scraped on the clean copper foil. Then, the copper foil loaded with slurry was first dried in a drying oven at 60 °C for 4 h; it was then transferred to a vacuum drying oven at 90 °C for 12 h. Finally, the dried electrode was cut into discs with a diameter of 12 mm by a punching machine, and then collected and labeled for later use.

Electrochemical measurements: The resulting product was used as a working electrode, with glass fiber (Whatman GF/D) as a separator and potassium foil as the reference electrode. The electrolyte composition was 0.8 M KPF_6_ in ethylene carbonate (EC)/diethyl carbonate (DEC) (*v*/*v* = 1:1). The CR2016 half-cells were assembled in a glove box filled with argon gas (H_2_O < 0.1 ppm, O_2_ < 0.1 ppm). A constant current charge–discharge cycle test was performed on the LAND battery test system (Wuhan Lande Electronics Co., Ltd., Wuhan, China) at room temperature (30 °C), and the potential window was 0.01–3.0 V. A CHI660d electrochemical workstation was used for cyclic voltammetry (CV) testing with a potential window of 0.01–3.0 V.

## 4. Conclusions

In summary, the ultra-thin 2D wrinkled soft carbon sheet prepared by using the template effect of MA was investigated as an anode material of PIBs. Due to their flexible and adjustable micro-structure, USCs manifest high specific capacity, excellent rate capability, and a long cycle life, showing fascinating application prospects in high-power scenarios. Both the thickness and crystal structure of USCs can be easily adjusted to regulate K^+^ storage behavior and optimize K^+^ transport kinetics. Especially, USCs electrode materials fulfill both high power and long service life requirements. The wrinkles on the micron-sized sheets allow ample space for volume expansion during K^+^ insertion, boosting the cycling stability of USCs. It is believed that USCs have taken a critical step in the development of anode materials for high-power and long-life PIBs.

## Data Availability

The data presented in this study are available in supplementary material.

## Supplementary Materials

The following supporting information can be downloaded at: https://www.mdpi.com/article/10.3390/molecules27092973/s1, Figure S1: SEM images of USC_10−1_; Figure S2: The CV curves at 0.1mV s^−1^ scanning speed and charge-discharge curves at 25 mA g^−1^: (a,b) USC_10−1_, (c,d) USC_50−1_; Figure S3: (a,e,i,m) CV curves at different scan rates, (b,f,j,n) linear fitting relationship between log i and log v at different redox peaks, (c,g,k,o) CV curves of electrode capacitance contributions at a scan rate of 1.0 mV s^−1^, (d,h,l,p) contribution ratio of pseudocapacitive response at different scan rates.
