# Supplementary material for: Ultra-Thin Wrinkled Carbon Sheet as an Anode Material of High-Power-Density Potassium-Ion Batteries
Source: Molecules. 2022 May 6;27(9):2973. doi: 10.3390/molecules27092973 (PMC9099802; doi:10.3390/molecules27092973)
Supplement: Supplementary file 1 [file molecules-27-02973-s001.zip › molecules-1648984-supplementary.pdf]

# Supporting information

# Ultra-Thin Wrinkled Carbon Sheet as an Anode Material of High-Power-Density Potassium-Ion Batteries

**Boshi Cheng [1,2], Xing Li [1,2,*], Linhai Pan [2], Hongqiang Xu [2], Haojie Duan [2], Qian Wu [2], Bo Yin [2,*] and Haiyong He [2]**

[1] School of Materials Science and Chemical Engineering, Ningbo University, Ningbo 315211, China; chengboshi@nimte.ac.cn

[2] Ningbo Institute of Materials Technology and Engineering, Chinese Academy of Sciences, Ningbo 315201, China; panlinhai@nimte.ac.cn (L.P.); xuhongqiang@nimte.ac.cn (H.X.); duanhaojie@nimte.ac.cn (H.D.); wuqian20@nimte.ac.cn (Q.W.); hehaiyong@nimte.ac.cn (H.H.)

* Correspondence: lixing@nbu.edu.cn (X.L.); yinbo@nimte.ac.cn (B.Y.)

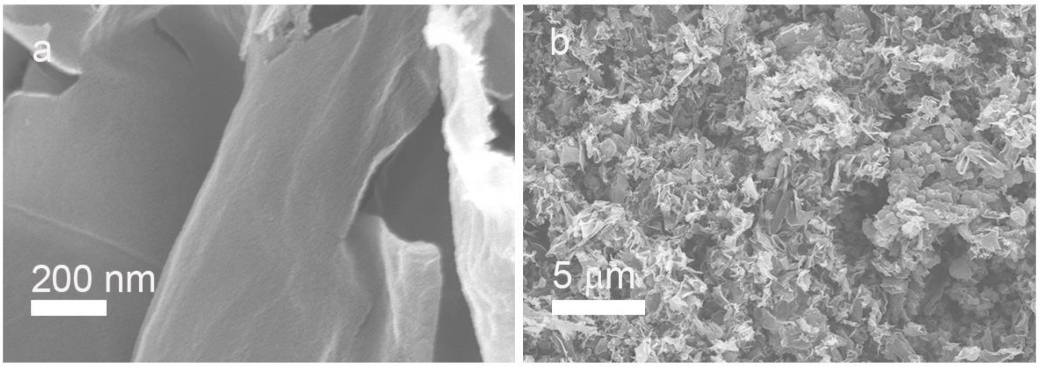


**Figure S1.** SEM images of $USC_{10-1}$.

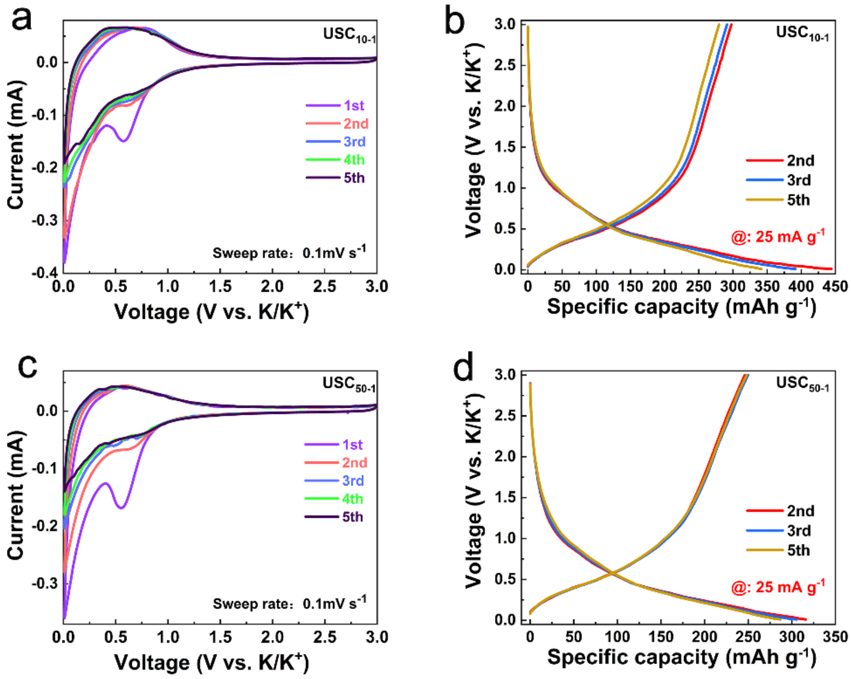


**Figure S2.** The CV curves at 0.1mV $s^{-1}$ scanning speed and charge-discharge curves at 25 mA $g^{-1}$: (**a,b**) $USC_{10-1}$, (**c,d**) $USC_{50-1}$.

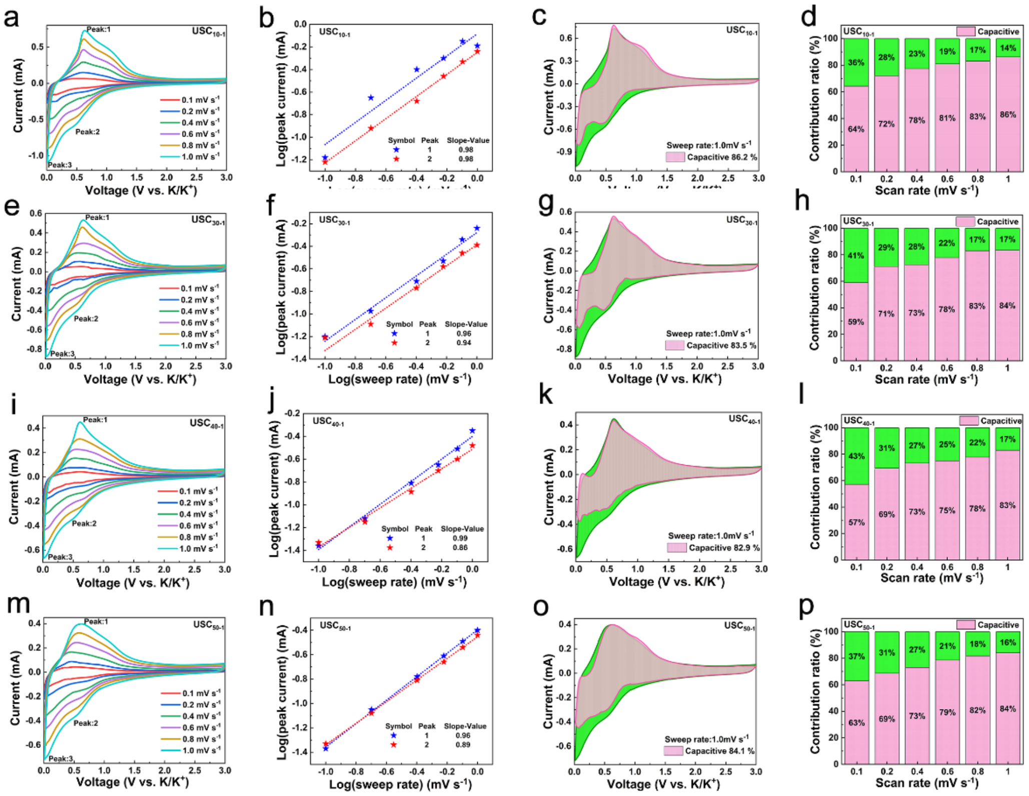


**Figure S3.** (**a, e, i, m**) CV curves at different scan rates, (**b, f, j, n**) linear fitting relationship between log i and log v at different redox peaks, (**c, g, k, o**) CV curves of electrode capacitance contributions at a scan rate of 1.0 mV $s^{-1}$, (**d, h, l, p**) contribution ratio of pseudocapacitive response at different scan rates.
